# Interleukin‐1β exacerbates disease and is a potential therapeutic target to reduce pulmonary inflammation during severe influenza A virus infection

**DOI:** 10.1111/imcb.12459

**Published:** 2021-05-20

**Authors:** Abdulah OS Bawazeer, Sarah Rosli, Christopher M Harpur, Callum AH Docherty, Ashley Mansell, Michelle D Tate

**Affiliations:** ^1^ Centre for Innate Immunity and Infectious Diseases Hudson Institute of Medical Research Clayton VIC Australia; ^2^ Department of Molecular and Translational Sciences Monash University Clayton VIC Australia; ^3^ King Faisal Medical City for Southern Regions Abha Saudi Arabia

**Keywords:** Disease, IL‐1β, inflammation, influenza A virus

## Abstract

Hyperinflammatory responses including the production of NLRP3‐dependent interleukin (IL)‐1β is a characteristic feature of severe and fatal influenza A virus (IAV) infections. The NLRP3 inflammasome has been shown to play a temporal role during severe IAV immune responses, with early protective and later detrimental responses. However, the specific contribution of IL‐1β in modulating IAV disease *in vivo* is currently not well defined. Here, we identified that activation of NLRP3‐dependent IL‐1β responses occurs rapidly following HKx31 H3N2 infection, prior to the onset of severe IAV disease. Mature IL‐1β was detectable *in vivo* in both hemopoietic and nonhemopoietic cells. Significantly, therapeutic inhibition of IL‐1β in the airways with intranasal anti‐IL‐1β antibody treatment from day 3 postinfection, corresponding to the onset of clinical signs of disease, significantly prolonged survival and reduced inflammation in the airways. Importantly, early targeting of IL‐1β from day 1 postinfection also improved survival. Together, these studies specifically define a role for IL‐1β in contributing to the development of hyperinflammation and disease and indicate that targeting IL‐1β is a potential therapeutic strategy for severe IAV infections.

## Graphical abstract

There is an urgent need to develop new drugs that limit damaging inflammation during severe influenza virus infection. Here, we illustrate that interleukin‐1β responses are rapidly activated in the lung during severe influenza virus infection, promoting hyperinflammation and disease development. Our data highlight the therapeutic potential and efficacy of targeting interleukin‐1β to limit severe influenza A virus infection.

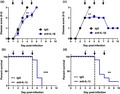

## INTRODUCTION

The coronavirus disease 2019 (COVID‐19) pandemic has demonstrated the catastrophic impact the emergence of a novel virus can have on human health, economic stability and how ill‐prepared we are in responding to an emerging pandemic. The threat that other pandemic viruses such influenza A virus (IAV) will emerge is a real and ongoing concern as recognized by the World Health Organization. Pathogenic avian H7N9 IAV infections in humans are currently associated with mortality rates of approximately 40% and experts predict a pandemic is inevitable.^1^

A characteristic feature of severe and fatal IAV infections is hyperinflammation, distinguished by excessive pulmonary immune cell infiltration and aberrant production of proinflammatory cytokines known as “cytokine storm” that contributes to lethality.^2^, ^3^ New and effective drugs for influenza and emerging viral pandemics are desperately needed to reduce hyperinflammation and the development of fatal disease.

NLRP3 inflammasomes are innate cytoplasmic complexes that are activated during IAV infection to enzymatically mature the inactive precursor cytokines, pro‐interleukin (IL)‐1β and pro‐IL‐18, into their bioactive forms IL‐1β and IL‐18. During IAV infection, activation of the NLRP3 inflammasome requires two signals.^4^ Signal one constitutes activation of the prototypic inflammatory transcription factor nuclear factor‐κB (NF‐κB) in response to pattern‐recognition receptor ligation which upregulates synthesis of pro‐IL‐1β, pro‐IL‐18 as well as components of the NLRP3 inflammasome such as NLRP3 and caspase 1. The second signal triggers inflammasome formation and involves a number of cellular processes or stimuli such as extracellular ATP activation of the P2X7 receptor^5^ and IAV proteins PB1‐F2 and M2,^6^, ^7^ leading to IL‐1β and IL‐18 maturation and secretion, as well as Gasdermin D cleavage and pyroptotic cell death.^8^

We have previously demonstrated that the NLRP3 inflammasome plays a temporal role during severe IAV infection.^9^ In particular, direct inhibition of NLRP3 from day 3 following the development of severe disease prolonged the survival of mice and limited hyperinflammation in the airways. However, treatment of mice from day 1 postinfection with the highly efficacious small‐molecule NLRP3 inhibitor MCC950 rendered mice more susceptible to infection, raising safety concerns regarding potent ablation of NLRP3 responses as a therapeutic strategy.

Currently, the specific *in vivo* role of IL‐1β in IAV pathogenesis is not well defined. IL‐1β acts downstream of NLRP3 following binding to the IL‐1 receptor (IL‐1R) to potently induce NF‐κB‐dependent inflammation, inducing immune cell trafficking (e.g. neutrophils and T cells), activation of epithelial and endothelial cells as well as autocrine/paracrine cytokine production [e.g. IL‐1β, tumor necrosis factor‐α (TNF‐α) and IL‐6].^10^, ^11^ Elevated levels of IL‐1β, TNF‐α and IL‐6 are prognostic markers of poor clinical outcomes during severe H7N9 IAV infections in humans.^12^, ^13^, ^14^ IL‐1β expression has also been shown to correlate with H1N1 disease in children.^15^, ^16^ In addition, mice lacking the IL‐1R in which both IL‐1α and IL‐1β signal have been shown to be more susceptible to infection with mouse‐adapted A/Puerto Rico/8/34 (PR8; H1N1) IAV.^17^, ^18^ However, reduced lung immunopathology was also observed in these animals, suggesting that IL‐1 signaling may play both protective and detrimental roles. In this study, we sought to specifically limit IL‐1β during severe IAV infection and to gain a greater understanding of the kinetics of tissue and cellular IL‐1β responses *in vivo*.

We report that rapid activation of IL‐1β responses in the lung following infection correlates with the development of severe disease during HKx31 H3N2 infection. Maturation of IL‐1β was observed in both hemopoietic and nonhemopoietic cells *in vivo* on day 1 postinfection. Inhibition of IL‐1β in the airways prior to/or following the development of severe disease limited hyperinflammation and prolonged survival, indicating that IL‐1β exacerbates disease and is a therapeutic target for severe IAV infection.

## RESULTS

### Kinetics of NLRP3 inflammasome expression during influenza virus infection

**Figure 1:**
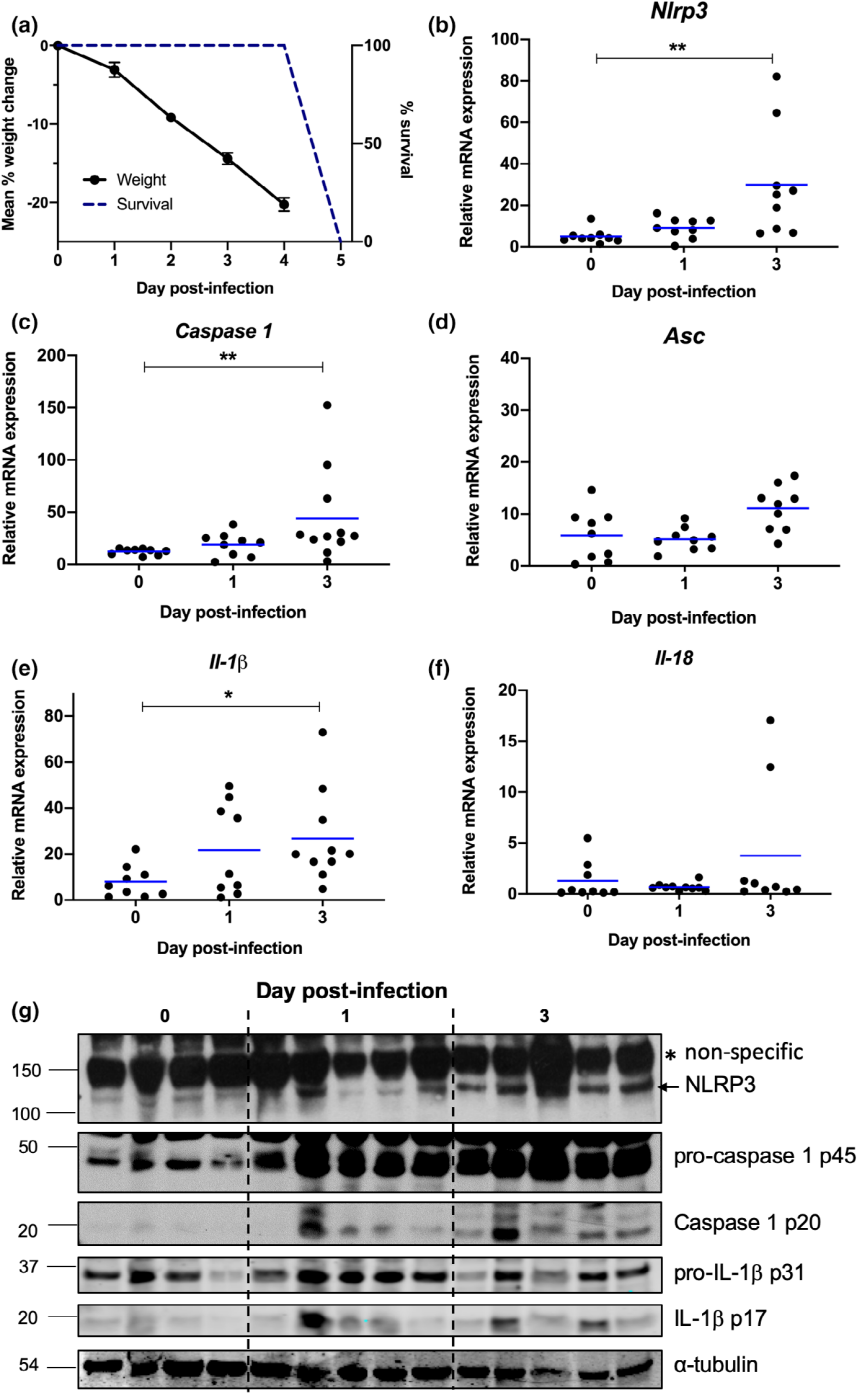
NLRP3 inflammasome responses and IL‐1β production in the lung during severe IAV infection. **(a–g)** C57BL/6 mice were infected with 10^5^ PFU of HKx31 as a model of severe IAV infection. **(a)** Percentage weight loss (mean ± s.e.m.) and survival of infected mice. **(b‐g)** Lungs were harvested from uninfected mice (day 0) and IAV‐infected mice on days 1 and 3 postinfection. mRNA expression of **(b)** *Nlrp3*, **(c)** *Caspase* *1,* **(d)** *Asc*, **(e)** *Il‐1β* and **(f)** *Il‐18* relative to *Gapdh*. Individual mice are shown as symbols and bars represent the mean. *P < 0.05, **P < 0.01; one‐way ANOVA. Data are representative of two independent experiments which were pooled (*n* = 8 or 9). **(g)** Immunoblot of NLRP3 (118‐kDa band labeled with an arrow and high‐molecular weight nonspecific band with an asterisk), procaspase‐1 (p45), caspase 1 (p20), pro‐IL‐1β (p31), IL‐1β (p17) and α‐tubulin protein. Data are representative of two independent experiments each consisting of four or five mice per group. IAV, influenza A virus; IL, interleukin; mRNA, messenger RNA; PFU, plaque‐forming units.

We have previously shown the NLRP3 inflammasome activates and promotes hyperinflammation and disease during severe IAV infection^6^, ^9^; however, the kinetics of the NLRP3 response *in vivo* are not well characterized. Infection of mice with 10^5^ PFU of the IAV strain HKx31 (H3N2) results in severe disease including rapid weight loss, reduced mobility and labored breathing requiring euthanasia on day 4 postinfection (Figure 1a).^5^, ^9^ To examine the kinetic expression of the NLRP3 inflammasome, C57BL/6 mice were infected with 10^5^ PFU of HKx31, and messenger RNA (mRNA; Figure 1b‐f) and protein (Figure 1g) expression was assessed in the lung on days 1 and 3 postinfection. Untreated controls were included for comparison (day 0) and were not inoculated with phosphate‐buffered saline (PBS), as we have previously shown PBS treatment does not induce inflammation.^6^, ^7^, ^19^ Expression of the NF‐κB‐dependent genes *Nlrp3* (Figure 1b) and *caspase 1* (Figure 1c)^20^, ^21^ were significantly increased in the lung on day 3, correlating with the development of severe disease (Figure 1a). By contrast, expression of the gene encoding the inflammasome adaptor ASC, which is not transcriptionally regulated by pattern‐recognition receptor‐mediated NF‐κB activation (i.e. signal 1),^20^, ^22^ was not significantly altered by infection (Figure 1d). Interestingly, *Il‐1β* mRNA significantly increased on day 3 postinfection compared with uninfected controls (Figure 1e), whereas conversely, *Il‐18* expression remained largely unchanged (Figure 1f), suggesting differential regulation of these genes in the lung. Overall, no significant differences in gene expression were seen between days 1 and 3 postinfection, suggesting that resident cells may respond rapidly on day 1 and that the global expression may be maintained on day 3 by the infiltration of large numbers of leukocytes. Immunoblot analysis (Figure 1g) indicated IAV infection induced a progressive upregulation of NLRP3 (top panel), most notably between days 1 and 3 postinfection. Interestingly, while procaspase 1 and pro‐IL‐1β were detectable from both uninfected and IAV‐infected lung tissue, only expression of procaspase 1 increased rapidly from day 1 postinfection. Importantly, the mature forms of caspase 1 and IL‐1β (p20 and p17 subunits, respectively), which are indicative of inflammasome activation, were observed at days 1 and 3 postinfection. Taken together, these data indicate that NLRP3 inflammasome is activated to rapidly mature IL‐1β prior to the onset of severe IAV disease.

### Hemopoietic and nonhemopoietic cells contribute to IL‐1β production following IAV infection

**Figure 2:**
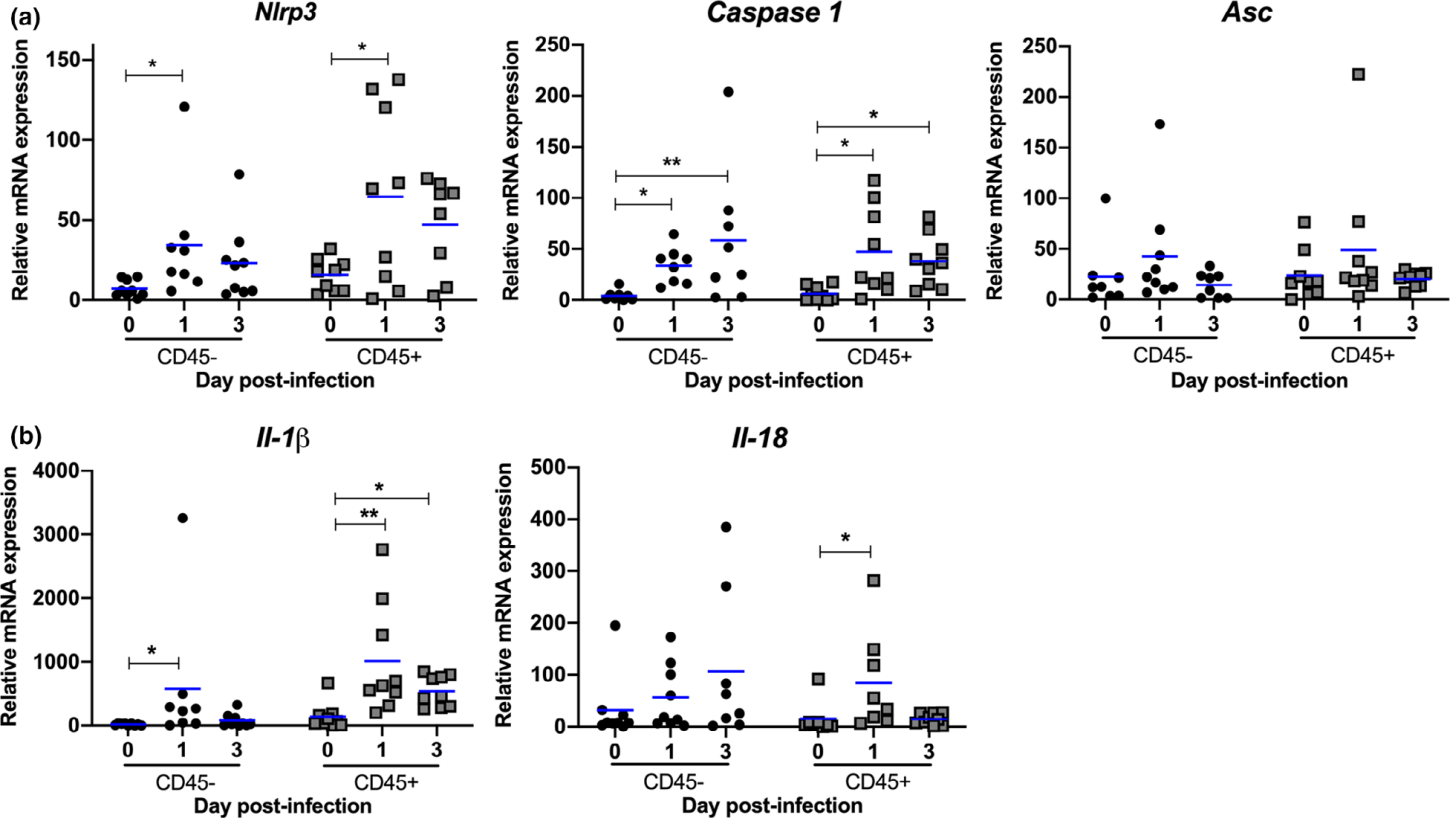
Hemopoietic and nonhemopoietic cells contribute to IL‐1β production following IAV infection. **(a, b)** C57BL/6 mice were infected with 10^5^ PFU of HKx31 and CD45^+^ hemopoietic and CD45^–^ nonhemopoietic cells were isolated from lungs on days 1 and 3 postinfection. Uninfected control mice were included for comparison (day 0). mRNA expression of **(a)** *Nlrp3*, *Caspase 1*, *Asc* and **(b)** *Il‐1β* and *Il‐18* relative to *Gapdh*. Data from two independent experiments were pooled (*n* = 8 or 9). Individual mice are shown as symbols and bars represent the mean. *P < 0.05, **P < 0.01; one‐way ANOVA. IAV, influenza A virus; IL, interleukin; mRNA, messenger RNA; PFU, plaque‐forming units.

Airway epithelial cells and resident alveolar macrophages are the primary targets of IAV infection.^23^, ^24^ In response to the infection, large numbers of leukocytes such as neutrophils and inflammatory macrophages infiltrate the airways from the blood.^9^, ^25^ To gain a greater understanding of the contribution of different cellular compartments to IL‐1β responses, we initially isolated CD45^+^ hemopoietic (e.g. macrophages, neutrophils) and CD45^–^ nonhemopoietic (e.g. epithelial cells) cells from the lungs of mice on days 1 and 3 postinfection (Figure 2). Expression of *Nlrp3* and *caspase 1* mRNA was significantly upregulated on day 1 post‐IAV infection in both CD45^–^ and CD45^+^ cells (Figure 2a). Consistent with our analysis of whole lung tissue (Figure 1d), *Asc* mRNA levels did not change significantly in either cell populations following IAV infection (Figure 2a). Interestingly, *Il‐1β* mRNA expression increased on day 1 postinfection in both cell populations; however, expression in CD45^–^ cells was transient (Figure 2b). In support of these findings, IL‐1β (p17) was detectable in both CD45^+^ and CD45^−^ isolated cells via immunoblot on day 1 (Supplementary figure 1). It is important to note, however, that equal protein loading was unable to be standardized as a result of sample protein concentrations falling below the detection level of a protein estimation assay and loading controls (α‐tubulin, glyceraldehyde 3‐phosphate dehydrogenase or β‐actin) not resolving on the reprobed immunoblots. By contrast, transcription of the *Il‐18* gene was not significantly altered following IAV infection in CD45^–^ cells, while a small but significant increase in *Il‐18* mRNA was observed on day 1 in CD45^+^ cells. Together, these results suggest that IL‐1β and IL‐18 expression is differently regulated in hemopoietic and nonhemopoietic cells within the lung during IAV infection.

**Figure 3:**
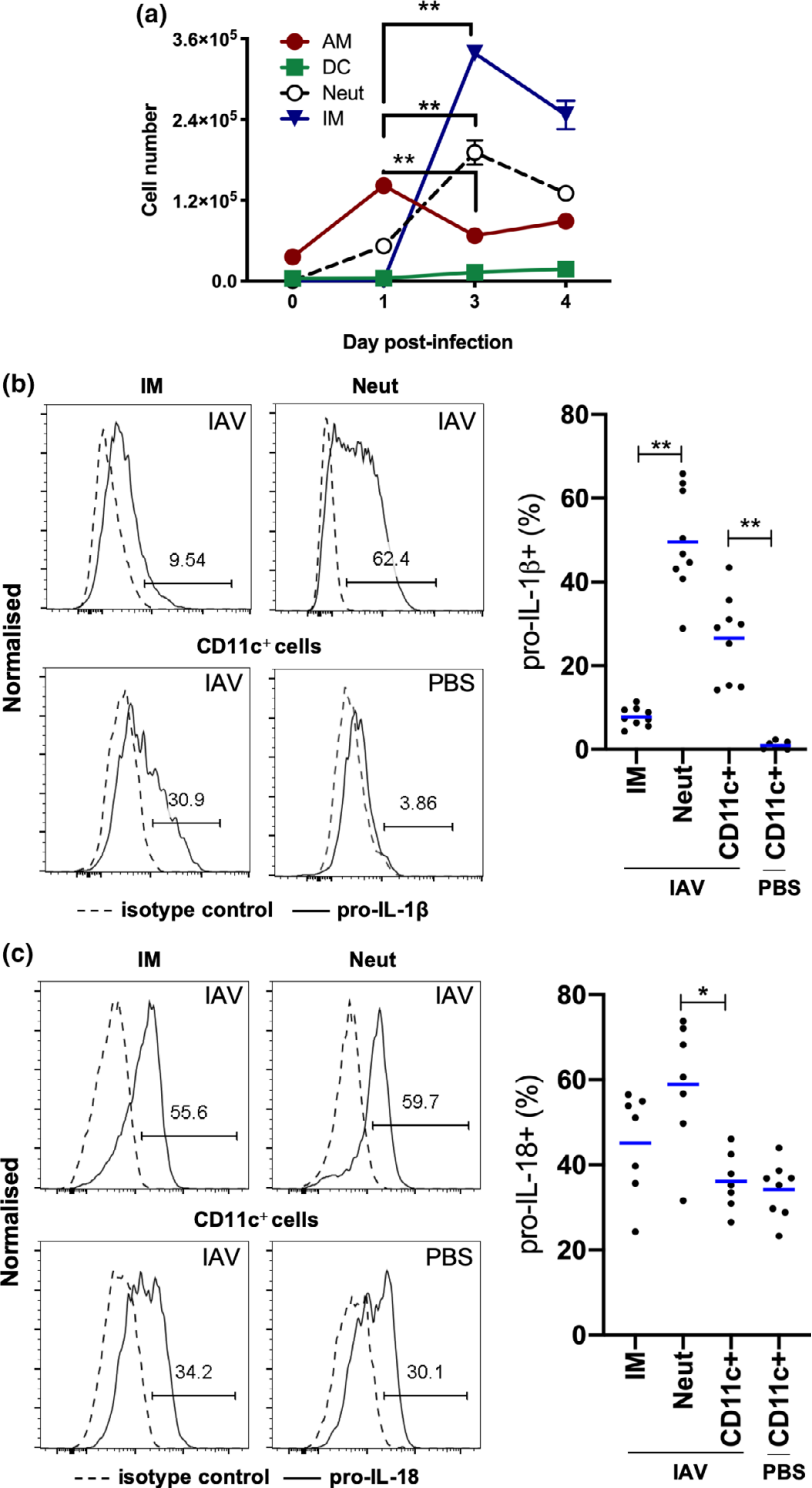
Expression of pro‐IL‐1β and pro‐IL‐18 by innate immune cells in BAL. **(a–c)** C57BL/6 mice were infected with 10^5^ PFU of HKx31 and BAL cells were isolated and characterized by flow cytometry. **(a)** Numbers of CD11c^+^I‐Ab^low^ AMs, CD11c^+^I‐Ab^high^ DCs, Ly6G^+^ neutrophils (Neut) and Ly6C^+^ IMs on days 0, 1, 3 and 4 postinfection. Mean ± s.e.m. **P < 0.01; day 1 *versus* day 3 only shown. **(b)** Representative histograms showing the expression of pro‐IL‐1β by BAL IMs, Neut and CD11c^+^ cells from uninfected PBS‐treated (PBS) or IAV‐infected mice on day 4. Proportion of pro‐IL‐1β^+^ IMs, Neut and CD11c^+^ cells in BAL. **(c)** Representative histograms showing expression of pro‐IL‐18 by BAL IMs, Neut and CD11c^+^ cells from uninfected PBS‐treated (PBS) or IAV‐infected mice on day 4. Proportion of pro‐IL‐18^+^ IMs, Neut and CD11c^+^ cells in BAL. Data are relative to isotype control. Individual mice are shown as circles and bars represent the mean. Neut and IMs were not analyzed in uninfected PBS‐controls because of low cell numbers. *P < 0.05, **P < 0.01; one‐way ANOVA. Data are from one of two experiments (*n* = 7 or 9). AM, alveolar macrophage; BAL, bronchoalveolar lavage; DCs, dendritic cells; IAV, influenza A virus; IL, interleukin; IM, inflammatory macrophage; mRNA, messenger RNA; PBS, phosphate‐buffered saline; PFU, plaque‐forming units.

To gain further insight into the expression of IL‐1β and IL‐18 in distinct leukocyte populations in the airways during IAV infection, we examined the intracellular expression of pro‐IL‐1β and pro‐IL‐18 in bronchoalveolar lavage (BAL) cells by flow cytometry. Airway macrophages reside in the airways in the absence of infection (day 0) with numbers increasing on day 1 postinfection, while numbers of dendritic cells remain relatively low throughout infection (Figure 3a). Neutrophils and Ly6C^+^ inflammatory macrophages were relatively absent in the airways of naïve mice but began to infiltrate within 1 day post‐IAV infection, reaching peak numbers in the BAL on day 3 postinfection (Figure 3a). IAV‐infected mice require ethical euthanasia on day 4 following infection and we next examined the intracellular expression of pro‐IL‐1β and pro‐IL‐18 at this timepoint. Intracellular expression of pro‐IL‐1β was increased in CD11c^+^ cells (i.e. airway macrophages and dendritic cells) in comparison to PBS‐treated uninfected controls (Figure 3b). Close to 50% of neutrophils expressed pro‐IL‐1β on day 4, compared with only approximately 8% of Ly6C^+^ inflammatory macrophages. By contrast, the proportion of intracellular pro‐IL‐18^+^CD11c^+^ cells did not change in response to IAV infection and approximately 50% of each cell type expressed pro‐IL‐18, including CD11c^+^ cells in the BAL of uninfected control mice (Figure 3c). Collectively, these results illustrate that pro‐IL‐1β and pro‐IL‐18 are expressed by CD11c^+^ cells, neutrophils and inflammatory macrophages in the airways of IAV‐infected mice at the peak of disease. In addition, the expression of pro‐IL‐1β and pro‐IL‐18 in CD11c^+^ macrophages and dendritic cells is differently regulated by IAV infection.

### IL‐1β exacerbates disease during severe IAV infection

**Figure 4:**
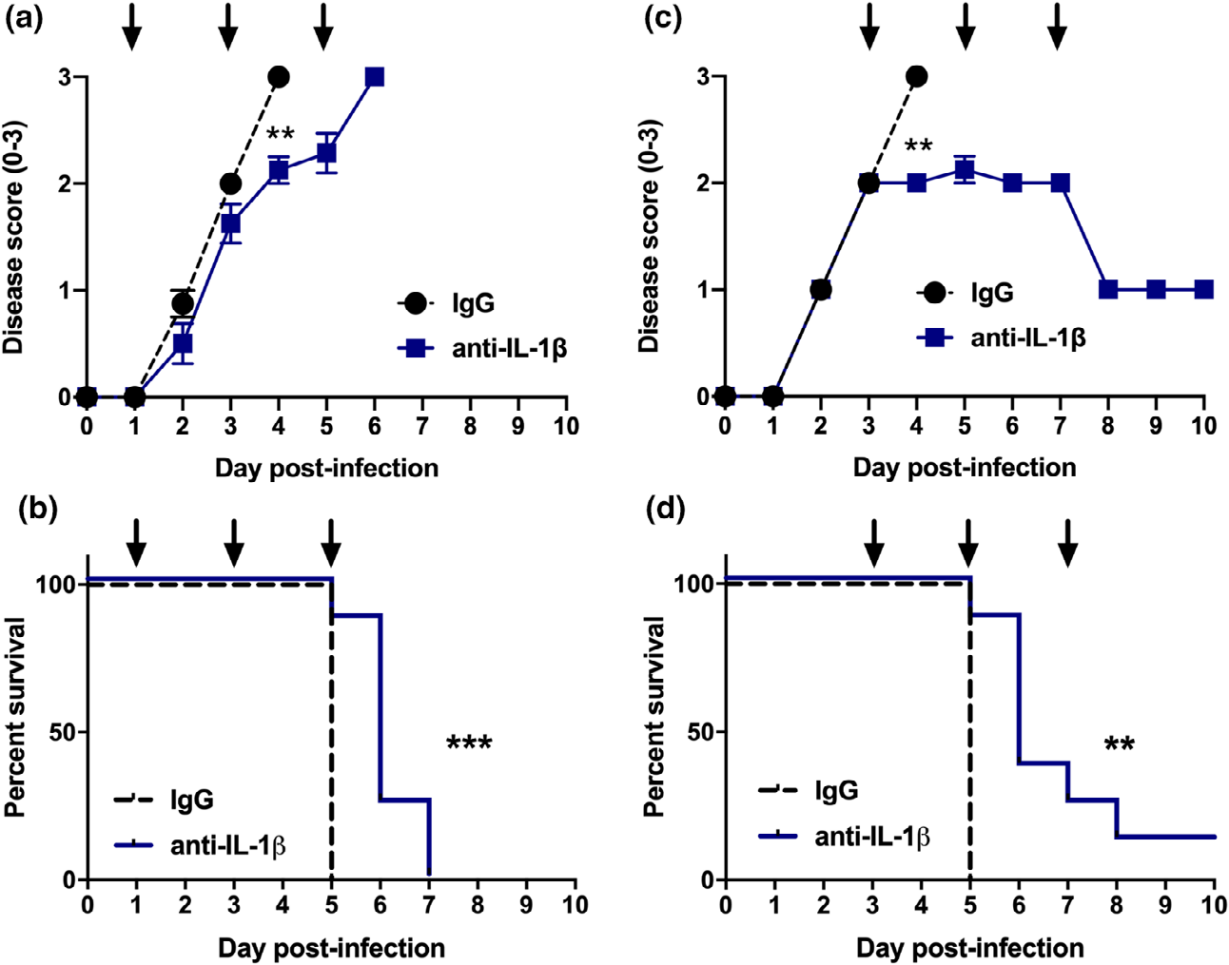
Anti‐IL‐1β antibody treatment prolongs the survival of IAV‐infected mice. **(a–d)** C57BL/6 mice were infected intranasally with HKx31 of 10^5^ PFU and intranasally treated with anti‐IL‐1β or control IgG antibodies. Antibody treatment was commenced on **(a, b)** day 1 or **(c, d)** day 3 following infection. Mice received additional treatments in 48‐h intervals as indicated with arrows. **(a, c)** Mice were assessed daily for clinical signs of disease (score of 0–3) as described in the “Methods” section. Data represent the mean ± s.e.m. **P < 0.01, Student’s *t*‐test. **(b, d)** Survival curves are shown. Mice displaying a clinical score of 3 (ruffled fur, reduced mobility or rapid breathing) or 20% or more weight loss were euthanized. **P < 0.01, ***P < 0.001, Mantel–Cox log‐rank test. **(a–d)** Data are from two independent experiments were pooled (*n* = 8). IAV, influenza A virus; Ig, immunoglobulin; IL, interleukin; PFU, plaque‐forming units.

Having established that maturation of IL‐1β correlates with the induction of severe IAV disease (Figures 1 and 2), we examined the role of IL‐1β at different stages of IAV infection. Mice were infected with 10^5^ PFU of HKx31 and intranasally treated with neutralizing anti‐IL‐1β or control IgG antibodies from day 1 or day 3 following infection. Mice received additional treatments every 48 h until they required ethical euthanasia or showed signs of recovery (e.g. weight gain; Figure 4). In contrast to our previous studies using the small‐molecule NLRP3 inhibitor MCC950,^9^ inhibition of IL‐β from day 1 (prior to the onset of severe disease) did not increase disease susceptibility, but rather delayed the onset of clinical symptoms such as hunched posture and reduced mobility (Figure 4a), and prolonged the survival of several mice by 1–2 days compared with IgG‐treated controls (Figure 4b). These results suggest that IL‐1β, in contrast to NLRP3, does not play an early protective role. Interestingly, commencing anti‐IL‐1β‐treatment on day 3 postinfection, closer to the onset of severe disease, limited the development of clinical symptoms (Figure 4c) and prolonged the survival of mice by up to 3 days with one of eight mice recovering (Figure 4d). Collectively, these results suggest that IL‐1β exacerbates disease during severe IAV infection and that early IL‐1β inhibition from day 1 does not render mice more susceptible to infection. Importantly, anti‐IL‐1β treatment initiated at the point of severe disease was at least as effective as starting prior to its onset, suggesting that targeting of IL‐1β can occur after the onset of symptoms and as such is a viable therapeutic option to improve severe IAV disease outcomes.

### IL‐1β inhibition at the onset of severe disease limits inflammation in the airways

**Figure 5:**
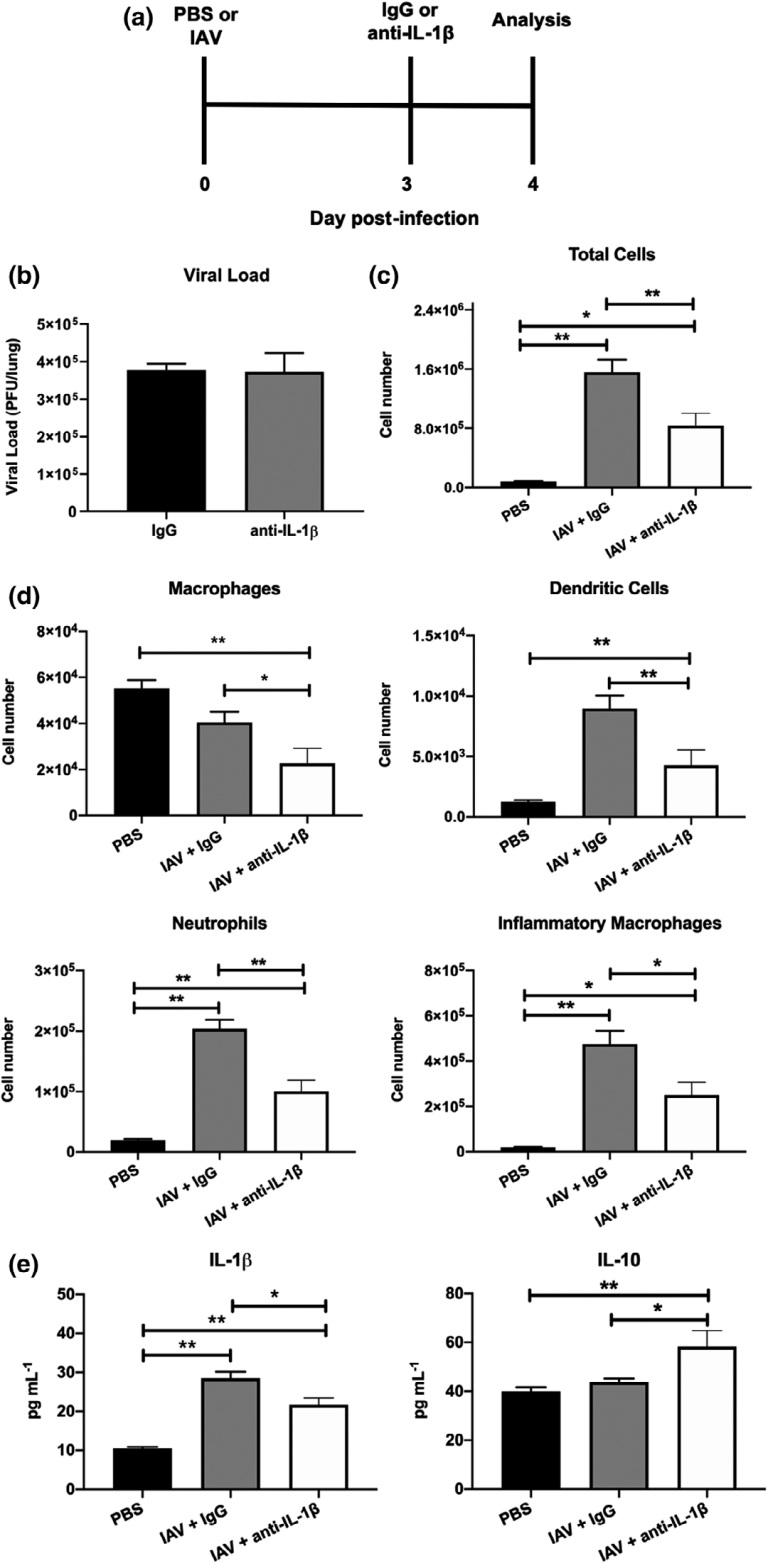
Anti‐IL‐1β antibody treatment modulates inflammation in the airways following IAV infection. **(a–e)** C57BL/6 mice were infected intranasally with 10^5^ PFU of HKx31 (IAV). On day 3 postinfection, mice were intranasally treated with anti‐IL‐1β or control IgG antibodies. Control uninfected mice were treated with PBS alone (PBS). On day 4 postinfection (i.e. 24‐h postantibody treatment), parameters of disease were examined. **(a)** Experimental timeline. **(b)** Viral loads in the lung determined by plaque assay. Not significant; *P* > 0.05; Student’s *t*‐test. **(c)** Total numbers of cells in the BAL determined by viable cell count. **(d)** Numbers of Ly6G^+^ neutrophils, CD11c^+^I‐Ab^high^ dendritic cells, CD11c^+^I‐Ab^low^ macrophages and Ly6C^+^ inflammatory macrophages in BAL determined by flow cytometry and viable cell counts. **(e)** Concentration of IL‐1β and anti‐inflammatory IL‐10 in BAL fluids determined by ELISA or cytometric bead array, respectively. **(a–e)** Data represent the mean ± s.e.m. Data from two independent experiments were pooled (*n* = 8). *P < 0.05, **P < 0.01; one‐way ANOVA. BAL, bronchoalveolar lavage; IAV, influenza A virus; IL, interleukin; PBS, phosphate‐buffered saline; PFU, plaque‐forming units.

Severe and fatal IAV infections are characterized by dysregulated cellular and proinflammatory cytokine responses.^2^, ^3^ Having established that inhibition of IL‐1β *in vivo* reduces severe IAV disease (Figure 4), we next examined the impact of intranasal anti‐IL‐1β antibody treatment on viral loads, cellular infiltrates and proinflammatory cytokines. As per Figure 4c, d, mice were treated with anti‐IL‐1β or control IgG antibodies on day 3 and analysis were performed 24 h post‐treatment (i.e. on day 4 or during the peak of disease; Figure 5a). Importantly, *in vivo* IL‐1β inhibition did not alter viral loads in the lung, suggesting that the previously observed effects on disease symptoms were not because of limiting viral replication (Figure 5b). Consistent with our previous studies with the NLRP3 inhibitor MCC950,^9^ treatment with anti‐IL‐1β antibodies significantly reduced total numbers of cellular infiltrates in the airways (Figure 5c), including macrophages, neutrophils, inflammatory Ly6C^+^ macrophages and dendritic cells in comparison to IgG‐treated controls (Figure 5d). Of note, anti‐IL‐1β treatment did not significantly alter cell frequencies (Supplementary figure 2a). As expected, levels of IL‐1β in BAL fluid were significantly reduced following anti‐IL‐1β antibody treatment (Figure 5e; *P* < 0.05). Levels of the proinflammatory cytokines IL‐6, TNF‐α (Supplementary figure 2b) and interferon‐γ (data not shown) were not significantly altered by the anti‐IL‐1β antibody treatment, while there was a slight, but not significant (*P* = 0.08), reduction in chemokine (C–C motif) ligand 2/monocyte chemoattractant protein‐1 (CCL2/MCP‐1) (Supplementary figure 2b). Interestingly, inhibition of IL‐1β significantly increased levels of the anti‐inflammatory cytokine IL‐10 (Figure 5e; *P* < 0.01). Overall, therapeutic targeting of IL‐1β during severe IAV disease reduced both inflammatory cellular infiltration of the lung and airway inflammation in the BAL.

## DISCUSSION

NLRP3 plays an initial early protective role during severe IAV infection^18^, ^26^, ^27^ but subsequently contributes to hyperinflammation and disease.^9^ The contribution of IL‐1β *in vivo* to IAV disease development and its potential as a therapeutic target warranted further investigation. In this study, IL‐1β responses were rapidly activated in the lung prior to the onset of severe IAV disease. Inhibition of IL‐1β *in vivo* demonstrated a clear detrimental role for IL‐1β in exacerbating the severity of IAV infection, including pulmonary inflammation. Importantly, in contrast to potent therapeutic inhibition of NLRP3 with the small‐molecule inhibitor MCC950,^9^ anti‐IL‐1β treatment from day 1 postinfection did not render mice more susceptible to IAV infection but rather improved survival. Collectively, these data highlight the therapeutic potential and efficacy of targeting IL‐1β in the early stages of severe IAV infection.

NLRP3 activation results in maturation of pro‐IL‐1β and pro‐IL‐18 into their bioactive forms IL‐1β and IL‐18, as well as pyroptotic cell death.^8^ Consistent with our results, anti‐IL‐1β treatment of mice following infection with the mouse‐adapted PR8 H1N1 strain was shown to broadly limit inflammation in lung tissue sections^28^; however, further detailed analysis was not performed by the authors. In contrast to our findings here demonstrating IL‐1β exacerbates IAV disease, studies in gene‐deficient mice have shown that IL‐18 plays a protective role during HKx31 and PR8 infection by limiting viral replication, mediating interferon‐γ production as well as NK and T‐cell responses.^29^, ^30^ Interestingly, we observed that *Il‐1β* and *Il‐18* mRNA were differentially regulated in the lungs of mice. IAV infection significantly increased *Il‐1β* mRNA expression in whole lung tissues. Expression of *Il‐1β* mRNA was also significantly upregulated by IAV infection in CD45^+^ hemopoietic (e.g. macrophages and neutrophils) and CD45^–^ nonhemopoietic cells (e.g. epithelial cells) isolated from lung tissues. By contrast, *Il‐18* mRNA was not significantly altered in whole lung tissues by IAV infection. We did observe a transient fivefold increase in expression of *Il‐18* in CD45^+^ leukocytes on day 1 postinfection, suggesting that neutrophils and inflammatory macrophages which infiltrate after day 1 may lowly express *Il‐18*. No significant changes in *Il‐18* were observed in CD45^–^ nonhemopoietic cells, potentially as a result of constitutive expression in these cells. In line with these results which suggest differential regulation of IL‐1β and IL‐18 expression, *Il‐1β* has been shown to be upregulated by NF‐κB,^31^ whereas the murine *Il‐18* promoter has been shown to contain an interferon consensus sequence‐binding protein, an activator protein‐1 and ETS transcription factor‐binding sites.^32^ Importantly, we observed mature IL‐1β in both hemopoietic and nonhemopoietic cells *in vivo* following IAV infection.

Severe and fatal IAV infections are characterized by the infiltration of high numbers of leukocytes. Treatment of mice with a single inoculation of anti‐IL‐1β antibody following the onset of clinical signs of severe disease (day 3) resulted in reduced numbers macrophages, neutrophils, inflammatory macrophages and dendritic cells in the airways 24 h thereafter. IL‐1β induces the secretion of NF‐κB‐dependent cytokines IL‐6, TNF‐α and CCL2; however, a single anti‐IL‐1β antibody treatment did not significant alter levels of these cytokines in BAL fluids. This result is not unexpected as a number of pathways regulate NF‐κB activity during IAV infection including those involving Toll‐like receptor 3 and 7, as well as retinoic acid inducible gene I (RIG‐I). Interestingly, inhibition of IL‐1β *in vivo* did result in a significant increase in the levels of the anti‐inflammatory cytokine IL‐10. While IL‐10 has been shown to inhibit IL‐1β responses,^33^, ^34^ to our knowledge this is the first report indicating a reciprocal negative feedback loop with IL‐1β negatively regulating IL‐10 expression. This observation warrants further investigation. In our study, mice were intranasally treated with anti‐IL‐1β antibodies every 48 h, resulting in prolonged survival, suggesting that the anti‐inflammatory effects of treatment are maintained for a minimum of 2 days. Further studies are required to assess the efficacy of delivering anti‐IL‐1β antibodies by other routes (e.g. intravenous and intraperitoneal), as well as differing treatment doses and intervals.

Mice lacking IL‐1R are more susceptible to PR8 infection,^17^, ^18^ suggesting that anti‐IL‐1β therapeutic strategies may be more advantageous than anti‐IL‐1R, as they have the potential to specifically target damaging immune responses without compromising important protective immune responses. Of note, therapeutic targeting of IL‐1R via intravenous treatment with recombinant IL‐1R antagonist from day 2 following infection was shown to improve survival^35^; however, inflammation in the lung was unfortunately not examined by the authors, nor the possibility that as per studies in IL‐1R^–/–^ mice, inhibition of IL‐1R from day 1 could result in increased susceptibility to infection. In contrast to our findings here with IL‐1β, Momota *et al*.^36^ recently illustrated that IL‐1α plays a protective role during PR8 infection. Specifically, IL‐1α was shown to be differentially regulated, with secretion of IL‐1α occurring independently of NLRP3/caspase 1 but rather resulting via DNA‐binding protein 1 (ZBP‐1)‐mediated cell death. In line with this study, administration of recombinant IL‐1α to the lungs of wild‐type mice early during HKx31 IAV infection was shown to promote germinal center B‐cell numbers, as well as tertiary lymphoid organ formation.^37^

Severe IAV and COVID‐19 infections share common pathological features such as the development of acute respiratory distress syndrome, characterized by pulmonary edema and vascular leakage.^3^, ^38^ Treatment of rats with IL‐1β as well as adenoviral overexpression of IL‐1β in mice has been shown to increase vascular permeability *in vivo*.^39^, ^40^, ^41^ IL‐1β has also been shown to inhibit fluid transport across the lung epithelium by decreasing the expression of the epithelial sodium channel α‐subunit.^42^ Canakinumab is an anti‐IL‐1β monoclonal antibody which binds and neutralizes human IL‐1β. Studies examining the ability of canakinumab to improve COVID‐19 disease are currently underway.^43^, ^44^ IL‐1β has also been implicated in the development of a number of respiratory diseases such as chronic obstructive pulmonary disease, silicosis and idiopathic pulmonary fibrosis.^45^, ^46^ In summary, our preclinical study indicates the therapeutic strategy of targeting IL‐1β during severe IAV infection and this warrants future clinical investigation.

## METHODS

### Influenza virus infection of mice and sample collection

Six‐ to eight‐week‐old male C57BL/6 mice were maintained in the specific pathogen‐free physical containment level 2 Animal Research Facility at the Monash Medical Centre, Melbourne, Australia. All experimental procedures were approved by the Animal Ethics Committee and experimental procedures carried out in accordance with approved guidelines.

The IAV strain used in this study was HKx31 (H3N2), which is a high‐yielding reassortant of A/PR/8/34 (PR8; H1N1) that carries the surface glycoproteins of A/Aichi/2/1968 (H3N2). Viruses were grown in 10‐day embryonated chicken eggs by standard procedures and titrated on Madin–Darby Canine Kidney cells. For virus infection studies, mice were lightly anesthetized with isoflurane and intranasally inoculated with 10^5^ PFU of HKx31 in 50 μL PBS, previously shown to induce severe disease in C57BL/6 mice.^5^, ^9^ At the time points indicated, whole lung tissues were removed and snap frozen in liquid nitrogen. Lungs were not perfused as we believed the impact of inclusion of low levels of circulating cells would be negligible. In some experiments, BAL fluid was immediately obtained by flushing the lungs three times with 1 mL of PBS and the remaining lung tissues were then removed and frozen immediately in liquid nitrogen. Titers of infectious virus in lung homogenates were determined by standard plaque assay on Madin–Darby Canine Kidney cells.

### Isolation of CD45^+^ and CD45^–^ cells from lung tissues following IAV infection

Whole lung tissues were harvested and digested in dissociation medium containing 1.5 mg mL^–1^ Pronase (Roche, Basel, Switzerland) and 0.1 mg mL^–1^ DNase I (Sigma Aldrich, St Louis, CA, USA) to generate single‐cell suspensions. Red blood cells were removed via treatment with red blood cell lysis buffer (Sigma Aldrich). Cells were stained with purified anti‐CD45 antibodies (clone 30‐F11; BD Biosciences, Franklin Lakes, NJ, USA) in PBS containing 2% fetal calf serum. CD45^+^ and CD45^–^ cells were separated with BioMag goat anti‐rat Ig‐coupled magnetic beads (Qiagen, Hilden, Germany). Isolated cell fractions were then equally divided for either mRNA or protein isolation for the analyses described below.

### RNA isolation and mRNA expression analysis

Whole lung tissues were excised from mice and immediately snap frozen in liquid nitrogen. Total RNA was extracted from lung tissues using TRIsure (Bioline, London, UK) followed by future isolation with the RNeasy mini kit (Qiagen), according to the manufacturer’s instructions. RNA was extracted from isolated CD45^+^ and CD45^−^ cell fractions using the RNeasy mini kit (Qiagen). RNA from lung tissues and isolated cells were DNase treated (Promega, Madison, WI, USA) and reverse transcribed to complementary DNA using Moloney murine leukemia virus reverse transcriptase (Promega), according to the manufacturer’s instructions. Real‐time quantitative PCR were performed with Power SYBR Green chemistry (Life Technologies, Carlsbad, CA, USA) on a QuantStudio 6 Flex (Applied Biosystems, Foster City, CA, USA). The mRNA expression of target genes was normalized to the housekeeping gene *Gapdh*. The following primer sequences were utilized: *Nlrp3* Forward CATTGGAGAAAATGCCTTGG, Reverse AAGTAAGGCCGGAATTCACC; *ASC* Forward *GAAGCTGCTGACAGTGCAAC,* Reverse *GCCACAGCTCCAGACTCTTC;* *Caspase 1* Forward CACAGCTCTGGAGATGGTGA, Reverse TCTTTCAAGCTTGGGCACTT; *Il1b* Forward CAACCAACAAGTGATATTCTCCATG, Reverse GATCCACACTCTCCAGCTGCA; *Il18* Forward GTTTACAAGCATCCAGGCACAG, Reverse GAAGGTTTGAGGCGGCTTTC; *Gapdh* Forward CATGGCCTTCCGTGTTCCTA, Reverse GCGGCACGTCAGATCCA.

### Examination of protein expression by immunoblot

Whole lung tissues were excised from mice and immediately snap frozen in liquid nitrogen. Lung tissues were homogenized in lysis buffer consisting of 250 mm Tris‐HCl (pH 6.8), 10% (w/v) sodium dodecyl sulfate, 20% (v/v) glycerol, supplemented with cOmplete protease inhibitor (Roche). Protein estimation was used for whole lung samples to normalize protein loading (Bio‐Rad DC Protein Assay, Hercules, CA, USA). Protein was isolated from CD45^+^ and CD45^–^ cell fractions with radioimmunoprecipitation (RIPA) assay buffer consisting of 50 mm Tris‐HCl (pH 8), 150 mm NaCl, 1 mm EDTA, 1% (v/v) IGEPAL, 0.5% (w/v) sodium deoxycholate, 0.1% (w/v) sodium dodecyl sulfate, 10 mm sodium fluoride, 1 mm sodium orthovanadate and 1 mm phenylmethylsulfonyl fluoride (Sigma Aldrich).

Protein lysates from whole lung tissues or CD45 cell fractions were resolved by 4–12% sodium dodecyl sulfate–polyacrylamide gel electrophoresis (Life Technologies), transferred onto PVDF (Merck Millipore, Burlington USA). The membranes were blocked with 5% bovine serum albumin (Sigma Aldrich) in Tris‐buffered saline with 0.1% Tween 20 (Sigma Aldrich) followed by incubation with the desired primary antibodies overnight: anti‐mouse caspase‐1 (clone Casper‐1; AdipoGen Life Sciences), anti‐mouse NLRP3 (clone Cyro‐2; AdipoGen Life Sciences), biotinylated anti‐mouse IL‐1β (clone 30311; R&D Systems, Minneapolis, MN, USA) or anti‐mouse α‐tubulin (clone YL1/2; Abcam). Membranes were probed with the appropriate secondary antibodies and visualized on the Bio‐Rad ChemiDoc MP Imaging System (Bio‐Rad, Hercules, CA, USA) via chemiluminescence (caspase 1, NLRP3) or fluorescence (IL‐1β, α‐tubulin). For CD45^+^ and CD45^–^ negative fractions, loading controls (α‐tubulin, glyceraldehyde 3‐phosphate dehydrogenase or β‐actin) did not resolve on reprobed blots; therefore, the expression of IL‐1β (p17) relative to pro‐IL‐1β (p31) was quantified using ImageJ software.

### Inhibition of IL‐1β *in vivo*

Mice were lightly anesthetized with isoflurane and treated via the intranasal route with 10 μg of rat IgG_1_ or anti‐IL‐1β (clone 30311; R&D Systems) antibodies in 50 μL of PBS, as previously described.^47^ For survival studies, antibody treatment was commenced on day 1 or 3 postinfection as indicated and mice received additional treatments every 48 h until they required ethical euthanasia or showed signs of recovery (i.e. weight gain). Mice were weighed daily and assessed for visual signs of clinical disease: 0 = no visible signs; 1 = slight ruffling of fur; 2 = ruffled fur, reduced mobility and 3 = ruffled fur, reduced mobility, rapid breathing. Animals that lost 20% or more of their original body weight or displayed a clinical disease score of 3 were euthanized.

### Quantification and characterization of leukocytes in the airways by flow cytometry

For the analysis of the effects of IL‐1β on cellular infiltration, mice were treated on day 3 following IAV infection with a single dose of rat IgG_1_ or anti‐IL‐1β (clone 30311; R&D Systems) antibodies in 50 μL of PBS. Control uninfected mice treated with PBS alone were included as controls. BAL cells were isolated on day 4 and incubated with Fc block (2.4G2; eBioscience, San Diego, CA, USA), followed by staining with fluorochrome‐conjugated monoclonal antibodies to Ly6C (clone AL‐21), Ly6G (1A8), CD11c (HL3) and I‐A^b^ (MHC‐II; AF6‐120.1). All antibodies were purchased from BD Biosciences, unless indicated. Neutrophils (Ly6G^+^), airway macrophages (CD11c^+^I‐Ab^low^), dendritic cells (CD11c^+^I‐Ab^high^) and inflammatory macrophages (Ly6G^−^Ly6C^+^) were quantified by flow cytometry, as described previously.^9^ Live cells were distinguished using propidium iodide (Sigma Aldrich) and compared with total viable cell counts, performed via trypan blue exclusion using a hemocytometer. The gating strategy for analysis is shown in Supplementary figure 3a.

For the analysis of intracellular staining of pro‐IL‐1β and IL‐18, BAL cells were isolated on day 4 postinfection. Uninfected PBS‐treated controls were included for comparison. BAL cells were fixed and permeabilized with Cytofix/Cytoperm kit (BD Biosciences) and stained with anti‐pro‐IL‐1β (clone NJTEN3; eBioscience), anti‐IL‐18 (clone 93‐10C; MBL International, Woburn USA) or isotype control antibodies (eBioscience). All antibodies were from BD Biosciences unless otherwise indicated. Samples were analyzed using the FACS Canto II or Fortessa X‐20 flow cytometer and FlowJo, version 10.7.1 (BD Biosciences). The gating strategy for analysis is shown in Supplementary figure 3b.

### Quantification of mouse proinflammatory cytokines in BAL fluid

BAL fluid was collected and stored at −80°C. IL‐1β was quantified by ELISA according to the manufacturer’s instructions (R&D Systems). Levels of IL‐6, interferon‐γ, TNF‐α, CCL2/MCP‐1, IL‐12p70 and IL‐10 were determined by cytokine bead array and mouse inflammation kit (BD Biosciences).

### Statistical analysis

When comparing three or more sets of values, a one‐way analysis of variance was used with Tukey’s *post hoc* analysis. A Student’s *t*‐test was used when comparing two values (two‐tailed, two‐sample equal variance). Survival proportions were compared using the Mantel–Cox log‐rank test. A *P*‐value < 0.05 was considered statistically significant.

## Author Contributions

**Abdulah OS Bawazeer**: Data curation; Formal analysis. **SarahRosli**: Data curation; Formal analysis; Investigation; Methodology; Supervision. **Christopher MHarpur**: Data curation; Formal analysis; Methodology; Writing‐review & editing. **Callum** **AH Docherty**: Data curation; Formal analysis. **Ashley Mansell**: Supervision; Writing‐review & editing. **Michelle D Tate**: Conceptualization; Data curation; Formal analysis; Funding acquisition; Investigation; Methodology; Project administration; Supervision; Writing‐original draft; Writing‐review & editing.

## CONFLICT OF INTEREST

The authors declare no competing financial interests.

## Data Availability

The data that support the findings of this study are available on request from the corresponding author. The data are not publicly available because of privacy or ethical restrictions.
