# Supplementary material for: Interleukin‐1β exacerbates disease and is a potential therapeutic target to reduce pulmonary inflammation during severe influenza A virus infection
Source: Immunol Cell Biol. 2021 May 20;99(7):737–48. doi: 10.1111/imcb.12459 (PMC8453884; doi:10.1111/imcb.12459)
Supplement: Supplementary file 1 [file IMCB-99-737-s001.pdf]

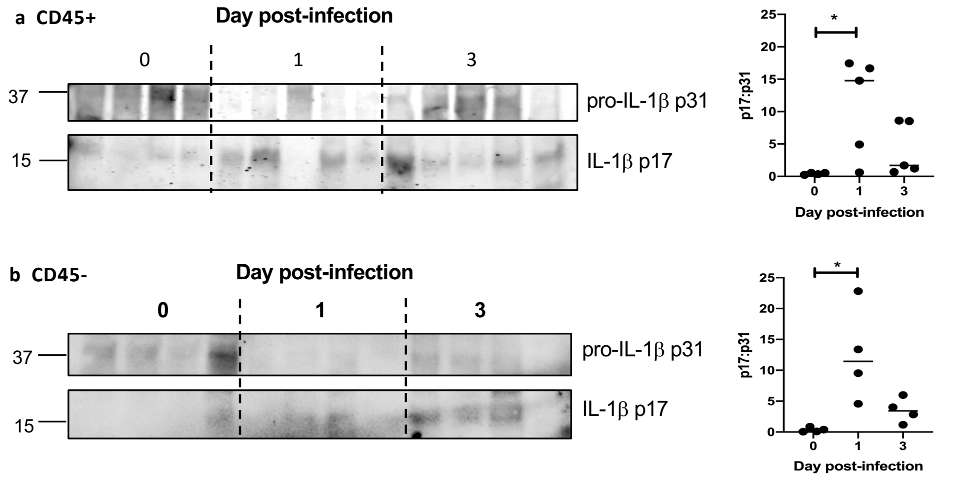

Supplementary Figure 1. (a-b) C57BL/6 mice were infected with 10^5 PFU of HKx31 and $CD45^+$ haemopoietic and $CD45^-$ non-haemopoietic cells were isolated from lungs on day 1 and 3 post-infection. Uninfected control mice were included for comparison (day 0). Immunoblot of pro-IL-1β (p31) and IL-1β (p17) in cell lysates from **(a)** $CD45^+$ and **(b)** $CD45^-$ cells. Loading controls did not resolve on the re-probed blots. Expression of p17 (IL-1β) relative to p31 (pro-IL-1β) is shown. * $P <$ 0.05; One-way ANOVA. Data are representative of 2 independent experiments each consisting of 4 or 5 mice per group.

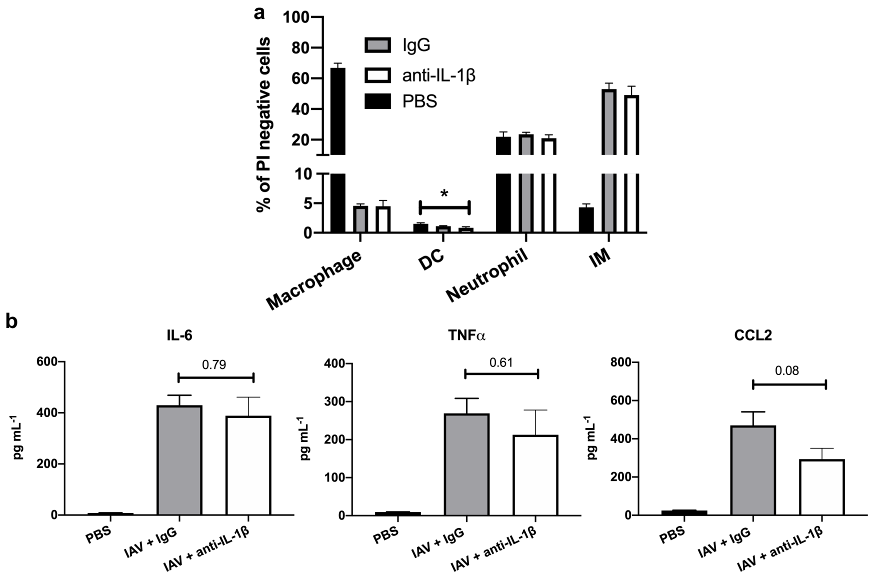

Supplementary Figure 2. (a-b) C57BL/6 mice were intranasally treated with anti-IL-1β antibody or IgG antibodies on day 3 following infection with 10^5 PFU of HKx31 (IAV). Control uninfected mice received intranasal PBS alone (PBS). On day 4 post-infection, disease parameters were examined. **(a)** Frequency of $Ly6G^+$ neutrophils, $Ly6C^+$ inflammatory macrophages (IM), $CD11c^+$ $I\text{-}A^b$ high dendritic cells (DC) and $CD11c^+$ $I\text{-}A^b$ low macrophages in BAL determined by flow cytometry. * $P < 0.05$, One-way ANOVA. **(b)** Concentration of IL-6, TNFα, CCL2 in in BAL fluids determined by cytokine bead array. *P*-values are shown IgG vs anti-IL-1β; One-way ANOVA. Levels of IL-6, TNFα, CCL2 were significantly increased in both IAV-infected groups in comparison to uninfected PBS controls ($P < 0.01$). **(a-b)** Data represents the mean ± SEM and is representative of 2 independent experiments which were pooled. n = 8.

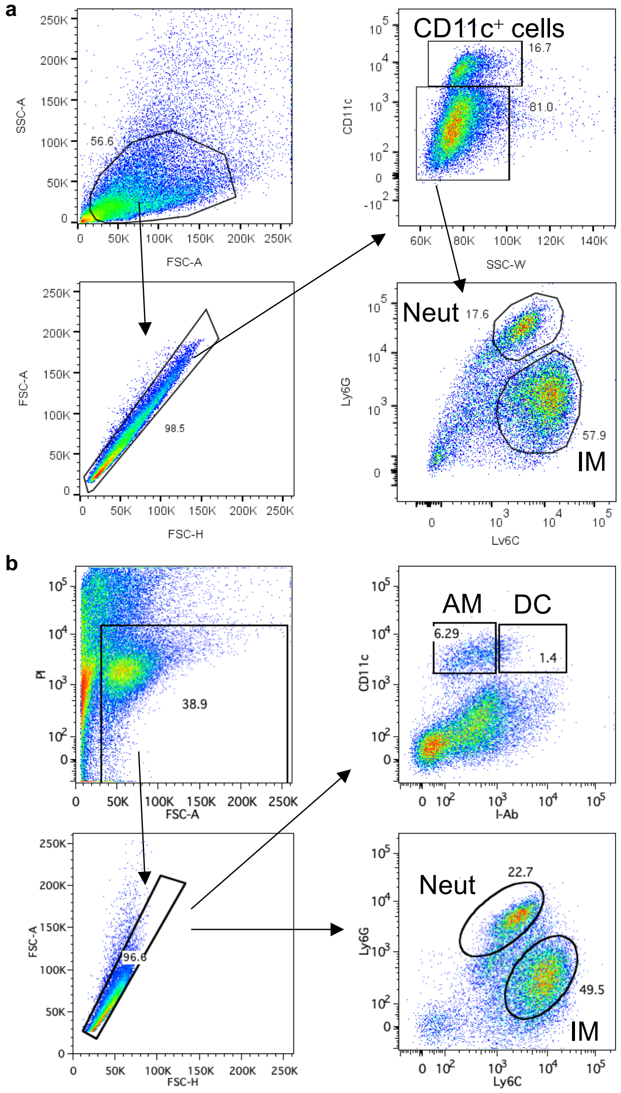

Supplementary Figure 3. (a) Flow cytometry gating strategy to examine intracellular pro-IL-1β and pro-IL-18 expression in CD11c^{+} cells, Ly6G^{+} neutrophils (Neut) and Ly6C^{+} inflammatory macrophages (IM) in the BAL. **(b)** Flow cytometry gating strategy to numerate numbers of PI^{-} CD11c^{+} I-A^{b} low alveolar macrophages, CD11c^{+} I-A^{b} high dendritic cells, Ly6G^{+} neutrophils and Ly6C^{+} inflammatory macrophages in the BAL.
